# Partial vision recovery after iatrogenic retinal artery occlusion

**DOI:** 10.1186/1471-2415-14-120

**Published:** 2014-10-11

**Authors:** Shu-Fang Hsiao, Yi-Hsun Huang

**Affiliations:** Department of Ophthalmology, National Cheng Kung University Hospital, College of Medicine, National Cheng Kung University, 138 Sheng Li Rd, Tainan, Taiwan

## Abstract

**Background:**

To describe the first case of partial vision recovery in a 32-year-old woman with iatrogenic retinal artery occlusion (RAO) following glabella calcium hydroxylapatite (CaHA) injection, and to explore appropriate diagnostic and therapeutic measures according to a literature review.

**Case presentation:**

A 32-year-old woman had left eye RAO and a bilateral visual field defect after CaHA injection into the glabella region. Topical and systemic intraocular pressure lowering agents, isovolemic hemodilution, globe massage, and anticoagulation with acetylsalicylic acid were prescribed. Carbogen inhalation and oral corticosteroids were also given. In addition to the above therapies, hyperbaric oxygen therapy (HBOT) was implemented as adjuvant treatment. The final best corrected visual acuity (BCVA) of the left eye improved from hand motion at 15 cm to 0.1. Improved retinal circulation and decreased retinal vessel leakage were found in the follow-up fluorescein angiography. However, there were still multiple emboli in the conjunctival and retinal arteries.

**Conclusion:**

This is the first case report on partial recovery of BCVA after iatrogenic RAO following cosmetic CaHA injection. Because no reliable treatments have been reported for such complications, HBOT may be considered as an alternative adjuvant therapy.

## Background

As soft-tissue augmentation of the face has become more common, there has been an increasing number of cases of ocular complications after facial filler injections, including hypersensitivity reactions, ecchymosis, anterior chamber inflammation, strabismus, ptosis, ophthalmoplegia, and retinal vessel occlusion [1]. Among these complications, permanent visual loss due to retinal artery occlusion (RAO) is the most devastating. To date, there have been no definite therapeutic guidelines developed for iatrogenic RAO. Although a few cases with vision improvement were reported in the past, [2–4] none of these cases were related to RAO following calcium hydroxylapatite (CaHA, RADIESSE®) injection. We herein describe a case of iatrogenic RAO following CaHA filler injection with vision and retinal circulation improvement after treatment. We used hyperbaric oxygen therapy (HBOT) as adjuvant treatment in this patient. To the best of our knowledge, this is the first case report of partial vision recovery in RAO following CaHA filler injection. We also provide a literature review to discuss the issues of diagnosis and treatment of this vision threatening disorder.

## Case presentation

**Figure 1:**
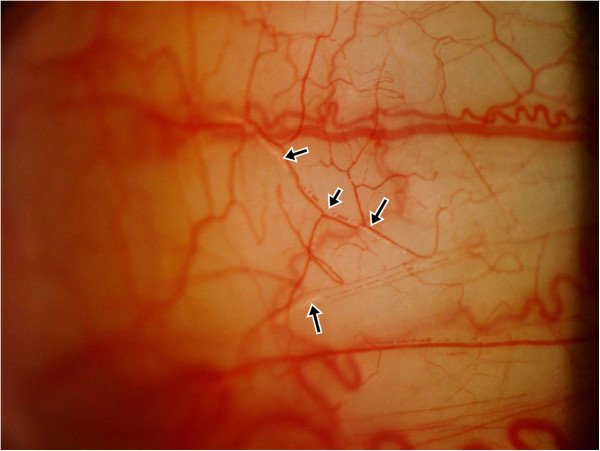
**Multiple emboli along the conjunctival vessels.**

**Figure 2:**
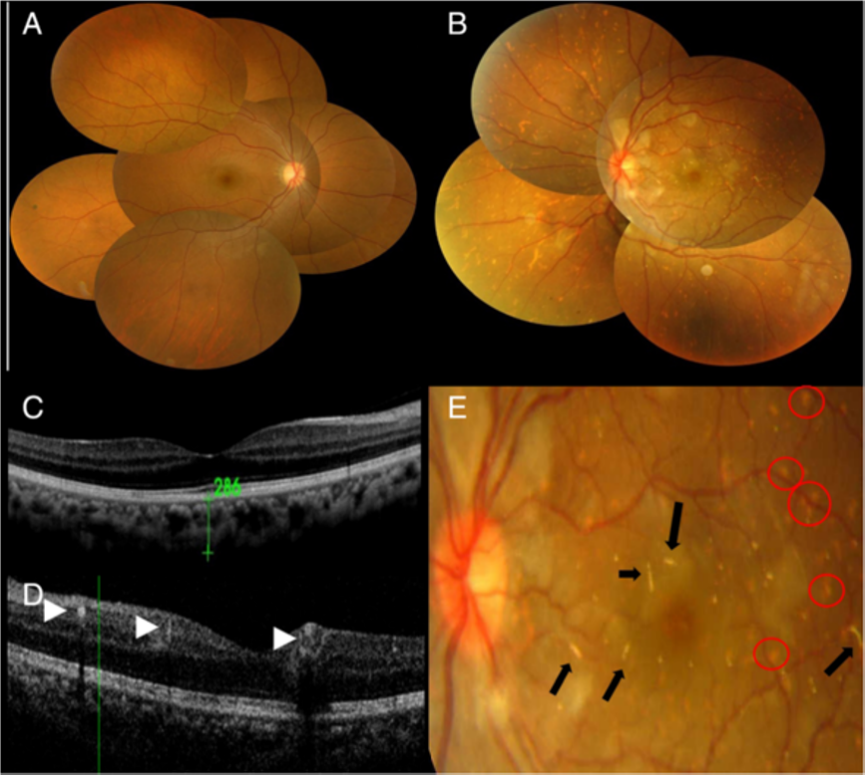
**Fundus picture. (A)** Normal in the right eye. **(B)** There were multiple emboli in the whole choroidal layer. **(C)** The corresponding SD-OCT in the right eye showed normal choroidal vascularity. **(D)** In the left eye, multiple hyper-reflective depositions (arrow head) were found in the retinal layer, which caused dark acoustic shadows, indicating CaHA emboli. **(E)** Magnification of the macular region shows multiple emboli (arrow) obstructing the distal retinal artery and choroidal layer (circle).

**Figure 3:**
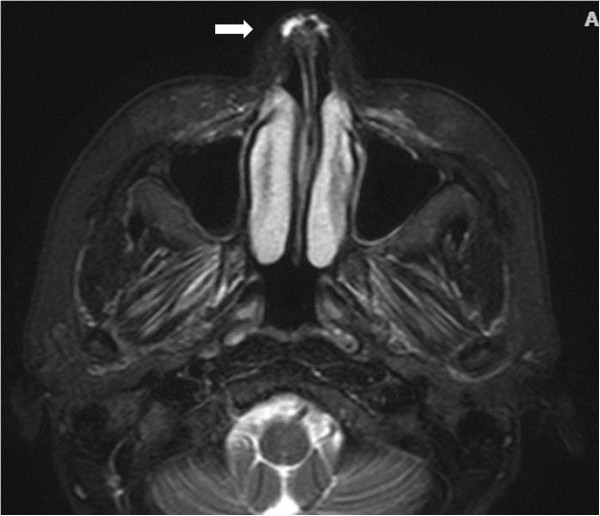
**T2W1MRI demonstrated high-attenuation material around the glabella region (arrow), corresponding with CaHA filler.**

**Figure 4:**
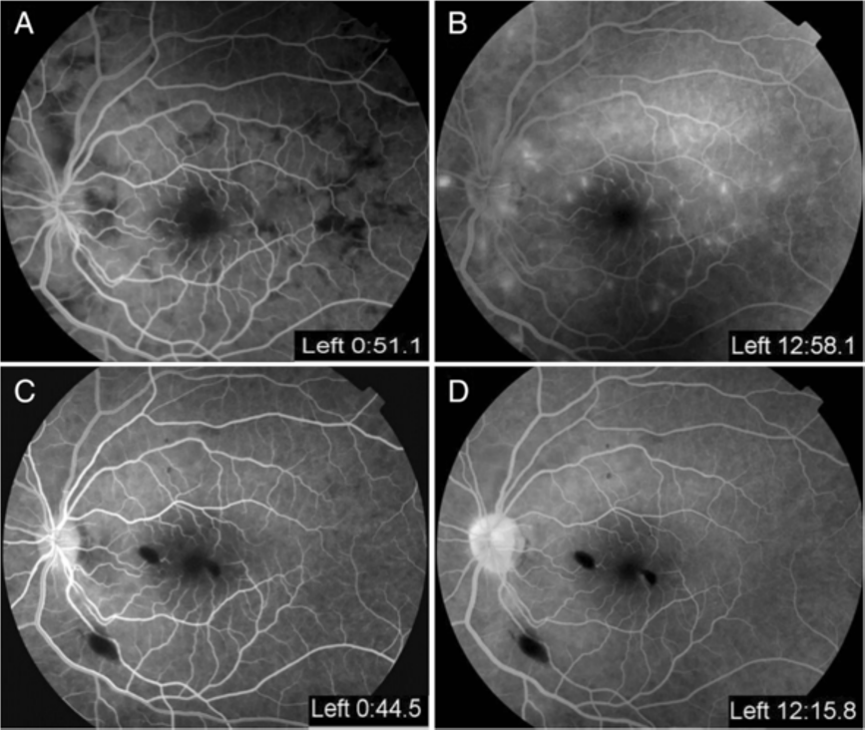
**Fundus fluorescein angiography (FAG) obtained on the second hospital day. (A)** Multiple absences of retinal perfusion in the arteriovenous phase. **(B)** Fluorescein leaking out of the occlusive retinal artery was found in the late phase. **(C)** FAG obtained 5 weeks later revealed recovery of retinal circulation, and **(D)** decrease retinal vessel leakage.

A 32-year-old woman sought treatment in the emergency department reporting sudden vision loss in her left eye. Four hours previously, she received vaginal plastic surgery and CaHA filler injection into the glabella region by a local plastic surgeon under general anesthesia. After waking up, she noted vision loss in her left eye, and was immediately transferred to our hospital. The initial best-corrected visual acuity (BCVA) at our emergency department was hand motion at 15 cm in the left eye and 1.0 in the right. The left pupil was dilated with a positive relative afferent papillary defect. There was neither ophthalmoplegia nor strabismus. Slit lamp examination showed multiple emboli along the conjunctival vessels (Figure 1). Fundoscopy in the right eye (OD) revealed normal findings (Figure 2A), while the left eye (OS) showed multiple emboli in the whole choroidal layer (Figure 2B). The corresponding spectral-domain OCT showed normal choroidal vascularity in the right eye (Figure 2C). In the left eye, multiple hyper-reflective depositions, which resulted in dark acoustic shadows (arrow head) in the retinal layer, were found (Figure 2D). Close-up imaging revealed distal RAOs (Figure 2E). Magnetic resonance imaging (MRI) showed no evidence of acute brain infarction, however, there were high-attenuation materials around the glabella region, which were compatible with the CaHA filler injections (Figure 3). Fundus fluorescein angiography (FAG) obtained on the second hospital day showed multiple absences of retinal perfusion in the arteriovenous phase (Figure 4A), and fluorescein leaking out of the occlusive retinal artery was found in the late phase (Figure 4B). Under the diagnosis of iatrogenic RAO, the therapies were administered to shorten the ischemic period on the basis of the European Assessment Group for Lysis in the Eye (EAGLE) guidelines, [5] including topical and systemic intraocular pressure lowering agents, isovolemic hemodilution, globe massage, and anticoagulation with acetylsalicylic acid. In addition, carbogen inhalation and oral corticosteroids were also provided.

**Figure 5:**
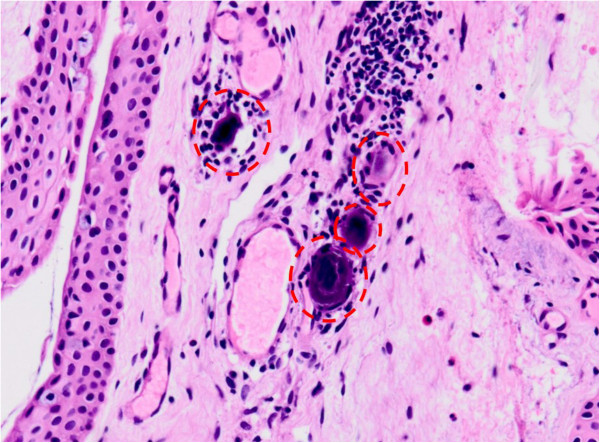
**The pathology of a conjunctival biopsy showed several calcified materials (dashed circle) in the capillary lumen.** Normal calcifications are not likely and foreign bodies are more likely to be present.

**Figure 6:**
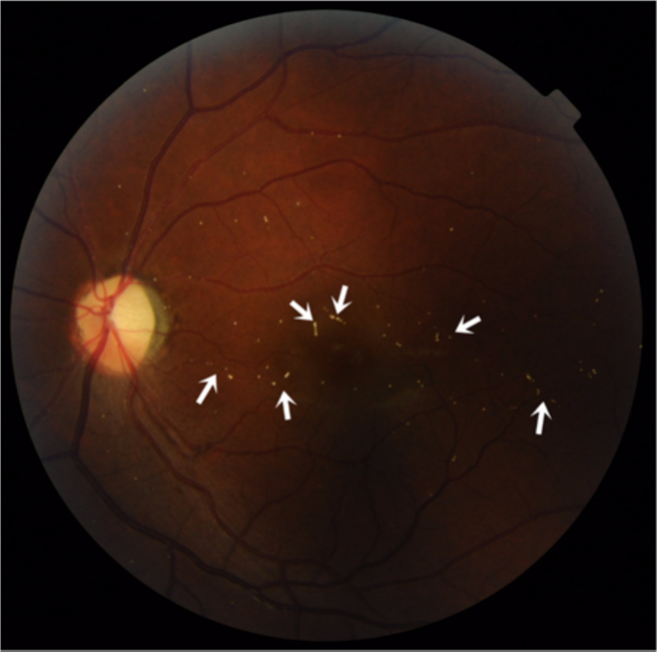
**Three months after treatment, the fundoscopic image of the left eye still showed multiple emboli in retinal vessels (arrow).**

Despite the treatment, progressive blurred vision was still found in the following few hours. Intra-arterial fibrinolysis using recombinant tissue plasminogen activator was not performed because of higher rates of adverse side effects [5]. An alternative treatment option, HBOT, was performed. Our patient received 100% oxygen via a face mask at a maximum ambient pressure of 2.5 atmospheres absolute. The protocol for the treatment was 10 minutes of compression, followed by a hyperbaric phase of 60 minutes, and 15 minutes of decompression. The treatment schedule was HBOT 3 times within the initial 24 hours, and 3 more treatments within the following 72 hours, for a total of 6 treatments. Conjunctival biopsy (OS) was performed later, which revealed multiple calcified foreign bodies in the vessel lumens (Figure 5). The visual acuity remained stable after the treatments, and the patient was discharged. Three months after onset, the BCVA improved from hand motion at 15 cm to 0.1 (OD). Fundoscopy in the left eye still showed multiple emboli in retinal vessels (Figure 6). Follow-up fluorescein angiography (FAG) showed improved retinal circulation (Figure 4C) and decreased retinal vessel leakage (Figure 4D).

### Discussion

Our literature review revealed that a retrograde embolic mechanism is generally thought to be the pathogenesis of RAO after cosmetic facial filler injections [4, 6]. Once an injection pressure is higher than the systolic arterial pressure, the injected material displaces the arterial blood and travels proximally past the origin of the retinal artery [7]. When the plunger is released, the arterial systolic pressure then propels the resulting column of material into the ophthalmic artery and its branches [6]. We searched PubMed and MEDLINE (1950 through February 2014) using the key words "visual loss", "visual impairment", "blindness", "retinal artery occlusion", "facial fillers", "facial injections", and "cosmetic procedure". Forty-six case reports of visual disturbance following facial filler injections were identified through the first screening [2–4, 6, 8–30]. We excluded the cases of visual disturbance not due to retinal artery occlusion after cosmetic injections. Only 2 cases met our criteria, which were related to CaHA filler injection [2, 9]. We summarized these 2 cases and made a comparison with our patient (Table 1).

**Table 1:** **Characteristics and clinical data of 3 patients with CaHA filler injection**

Case	Age (yr)	Sex	Eye	Injection site	Diagnosis	Associated ocular symptoms	Visit/Therapy interval	Treatment		Initial BCVA	Outcome		Follow - up
								EAGLE 6-step therapy	Adjunctive treatment		Final BCVA	Other ocular symptoms	
Sung MS, 2010 [2]	25	M	Right	glabella	PCAO, anterior segment ischemia	Dilated pupil, exotropia, ophthalmoplegia, hyphema, hypopyon, cornea edema, Ptosis	Immediately	One-step	Topical and systemic abx	HM	1.0	Resolved anterior segment inflammation	3M
Kim YJ 2013 [9]	30	M	Bilateral	glabella	Ophthalmic artery occlusion	Dilated pupil, total ophthalmoplegia, ptosis	NA	NA	NA	NLP	NLP	NA	NA
Current study	33	F	Bilateral	glabella	Retinal artery occlusion	Dilated pupil, RAPD, conjunctival injection	Immediately	Five-step	Carbogen, HBOT,	OD : 20/20 OS : HM	OD : 20/20 OS : 0.1	Recovery of choroidal and retinal circulation in the left eye	3M

abx = antibiotics; BCVA = best corrected visual acuity; EAGLE = European Assessment Group for Lysis in the EYE study; F = female; HBOT = hyperbaric oxygen therapy; HM = hand motion; M = male; NA = not available; NLP = negative light perception; PCAO = posterior ciliary artery occlusion; RAPD = relative afferent pupillary defect; yr = years.

Our case is the first case report of vision and circulation improvement in posterior segment ischemia due to CaHA filler injections. A variety of measures have been advocated in an attempt to treat non-arteritic retinal artery obstruction, but there were no generally agreed upon treatment regimens, with no suggested treatment regimens for iatrogenic RAO. In 2010, Sung et al. [2] first reported a case of anterior segment ischemia concurrent with vascular embolization after injection of CaHA. The main affected part was the anterior segment, and the involved vessel was the long posterior ciliary artery. Therapies included a topical antibiotic, topical steroids, and low-dose oral corticosteroids. The significant improvement in final visual acuity was determined by the resolution of corneal edema and severe anterior chamber inflammation, rather than an improvement in the posterior choroidal circulation. Theoretically, a limited nasal choroidal ischemia, sparing the macula, will not result in an obvious visual acuity disturbance. Kim and Chou reported a patient with bilateral blindness after CaHA injection in 2013 [9]. The main affected part was the posterior segment, and the involved vessel was the ophthalmic artery. However, only limited information on the treatments was provided, and the final visual outcome was no light perception in both eyes. In our case, the injured arteries were the retinal artery and short posterior ciliary artery. We provided the treatment immediately when the diagnosis was made. We completed 5 steps of a 6-step therapy, which followed the EAGLE study, [5] including topical and systemic lowering of intraocular pressure agents, ocular massage, hemodilution, and antiplatelet therapy with acetylsalicylic acid. In addition, interventions that had been used as treatments for acute retinal artery occlusion were provided at the same time, including carbogen breathing, and low dose oral prednisolone. Intra-arterial fibrinolysis using recombinant tissue plasminogen activator was not performed; instead, HBOT was implemented in 6 treatments. Three months later, the BCVA in the left eye increased from HM/15 cm to 0.1, and the follow-up FAG indicated recovery of retinal perfusion.

In our literature review of 46 case reports, 15 reported therapies for iatrogenic RAO, including dilation of the artery by breathing carbon dioxide/carbogen [20] and using an oral vasodilator, [19, 29] physical removal of the obstruction by ocular massage, [4, 20, 26, 27, 29] reduction of intraocular pressure by anterior chamber paracentesis, [4, 27] intravenous acetazolamide or mannitol, [3, 4, 10, 27, 29] intra-arterial thrombolysis, [4, 22] anti-platelet therapy, [10, 12, 19] reduced red blood cell rigidity by hemodilution, [20] and systemic steroids to reduce retinal edema [2, 11, 12, 21–24]. Only 4 of the 47 cases, including our case, showed improvements in visual acuity [2–4]. The number of cases was too small to provide a safe, convincing, and reliable treatment protocol for RAO. In our case, we observed a shorter occlusion time and faster restoration of perfusion. Thus, the rational goal of treatment should be shortening the occlusion time and increasing perfusion.

The major difference in our case from previous reports is the early HBOT within 24 hours after the onset of symptoms. A number of investigations have shown that early HBOT within 24 hours after the onset of symptoms had beneficial effects for visual acuity improvements in eyes with RAO, and there were no serious major complications [31–34]. However, for iatrogenic RAO, there have been no case reports or large clinical trials to prove the efficacy of HBOT. Breathing 100% oxygen under 2.5 atmospheres absolute can increase dissolved plasma oxygen by almost 17-fold than breathing room air. Under such hyperbaric conditions, enough dissolved plasma oxygen can meet the normal requirements of the body at rest without the need for hemoglobin [35]. Therefore, more oxygen can be delivered deeper into the ocular tissue, including the inner retina via diffusion, to support tissue survival until recovery of perfusion in patients with iatrogenic RAO [34]. Thus, though the CaHA particles still remained in place for a few months after occlusion, we surmised that the effect of HBOT in iatrogenic RAO could be the increase in oxygen perfusion into the retina, which further contributed to the improvement in visual acuity.

Another issue that needs to be mentioned is the possibility of central nervous system infarction. Just as the injected materials can be pushed into the ophthalmic artery, an excessive force may also cause retrograde emboli into the internal carotid artery, resulting in a cerebrovascular event. In our review, 7 of the 46 cases (7/46, 15.2%) [4, 10, 15, 17, 30, 36] had complications with a stroke event. Of these, 6 cases were following fat transfer, [4, 15, 17, 30, 36] and the remaining case had hyaluronic acid injection [10]. Therefore, we should pay attention to each patient’s neurological symptoms and signs. It is recommended that routine neuroimaging examination, such as CT or MRI, should be arranged in iatrogenic RAO to rule out the possibility of brain infarction.

## Conclusion

In conclusion, considering there are no reported reliable treatments for iatrogenic RAO, a poor visual acuity outcome is inevitable. In this report, we offer our experience with HBOT for the treatment of iatrogenic RAO. We found that early HBOT had some beneficial effects on retinal perfusion and visual acuity, while there were no major complications. Therefore, along with recommended treatments by the EAGLE study, [5] HBOT seems worthwhile for iatrogenic RAO within the initial 24 hours if the equipment and a trained technician are available.

### Consent

Written informed consent was obtained from the patient for publication of this Case Report and any accompanying images. A copy of the written consent is available for review by the Editor of this journal.

## Abbreviations

RAO: Retinal artery occlusion
FAG: Fluorescein angiography
OCT: Optical coherence tomography
MRI: Magnetic resonance imaging
HBOT: Hyperbaric oxygen therapy
BCVA: Best corrected visual acuity
CaHA: Calcium hydroxylapatite
EAGLE: European assessment group for lysis in the Eye.
